# No More Bricks in the Wall: Adopting Healthy Lifestyles through Physical Education Classes

**DOI:** 10.3390/ijerph16234860

**Published:** 2019-12-03

**Authors:** Rubén Trigueros, Adolfo J. Cangas, José M. Aguilar-Parra, Joaquín F. Álvarez, Alexandre García-Más

**Affiliations:** 1 Department of Language and Education, University of Antonio de Nebrija, 28015 Madrid, Spain; 2 Department of Psychology, University of Almería, 04120 Almería, Spain; ajcangas@ual.es (A.J.C.); jalvarez@ual.es (J.F.Á.); 3 Faculty of Psychology, GICAFE research group, University of the Balearic Islands, 07122 Palma, Spain; alex.garcia@uib.es

**Keywords:** physical education, physical activity, psychological well-being, healthy habits, adolescents

## Abstract

Despite the multiple benefits associated with practicing physical activity regularly, less than 20% of the population do it on a daily basis. Physical education classes could contribute, during childhood and adolescence, to consolidating adherence to healthy lifestyle habits. The present study involved 606 secondary school students between the ages of 13 and 19. We analysed the relationships between the perception of psychological control and support for autonomy, the satisfaction and frustration of psychological needs, mind-wandering and mindfulness, positive and negative emotions, motivation towards physical education classes, physical activity and the intention to be physically active—all through a structural equation model, which presented acceptable goodness-of-fit indices. The results showed that students who feel more autonomous see that their psychological needs are met and feel emotionally positive; this will result in the development of autonomous motivation towards physical education classes and physical activity that, in turn, could lead to a greater intention to be physically active.

## 1. Introduction

One of the fundamental objectives of physical education (PE) classes is for the students to adopt healthy lifestyle habits, as well as to participate in physical activity (PA) during their free time [1]. However, only 20% of adolescents around the world practice PA, despite the multiple physical benefits (decreased obesity, prevention of heart disease, etc.) and psychological benefits (prevention of stress, depression, and body image disorder, etc.) which daily practice provides [2]. It is therefore necessary to intervene so as to increase adolescent engagement in PA. To this effect, PE classes during primary and, especially, secondary education have been recognized as an ideal context in which to promote healthy physical-play activities during free time [3,4], in addition to improving physical abilities, and a wide range of competencies, such as cognitive and affective processes, motivation, and personal, social and emotional development. Here, the role of the PE teacher can be fundamental in developing these attitudes in students towards PA practice because of the influence he/she has on them, which comes from his/her knowledge of the subject and the daily interaction with students. Moreover, the emotional, cognitive, and motivational processes that take place during PE classes can be of great relevance; indeed, various studies in sports contexts consider them key to perpetuating certain behaviours such as dedication to training and continuing PA practice, etc. [5].

### 1.1. Self-Determination Theory

Self-determination theory (SDT, [6]) is a macro theory of human motivation that describes how human behaviour is influenced by the social and interpersonal environment. According to SDT, autonomous motivation is related to behaviours based on personal initiative, self-choice, and decision-making. Controlled motivation on the other hand is related to participation in events being due to external pressures or acquired obligations. This latter type of motivation leads to a lack of self-regulation regarding the adaptive behaviour as it tends to cease and move away from the action due to the absence of awards or external social recognition where they previously existed [7]. In contrast, autonomous motivation facilitates adaptation, leading to self-regulating behaviour because people tend to persist and adhere to them when they feel the enjoyment coming from personal improvement and the sensations originating from the activity itself. In this sense, SDT suggests that motivation has a psychological origin resulting from the interaction of three psychological needs—competence, relatedness, and autonomy—which are basic and universal in all human beings, and that are defined as essential nutriments for personal development and well-being [8], in such a way that, if satisfied, will give rise to autonomous motivation. Recently, in a study conducted by González-Cutre, Sicilia, Sierra, Ferriz, and Hagger [9], the incorporation of a fourth psychological need was proposed—novelty—which is linked to the need to experience something new or that deviates from the daily routine.

The PE context is one of the areas where SDT is most applied. It has been demonstrated how satisfying psychological needs leads to autonomous motivation, which is related to the learning of new skills, commitment to learning and improvement of interpersonal relationships [7]. Accordingly, students feel more autonomous when they participate in decision-making, feel competent when they perceive themselves effective at performing the task at hand, and have a sense of relatedness when they feel accepted, supported, and integrated into their social reference group [7]. Therefore, if the PE teacher is able to generate a social climate that favours meeting the basic psychological needs of students during PE classes, students will tend towards autonomous motivation when doing them. In addition, students may pursue similar behaviours in extracurricular environments to meet their basic psychological needs [10]. This itinerary through the SDT is usually known as the clear side, making a clear allusion to the positive side more adaptive [11].

However, these studies in the PE field have been carried out by looking at the motivational space of teacher autonomy as perceived by the student, with no evidence showing how the teacher’s controlling behaviours, as perceived by the student, influence their basic psychological needs [6]. Thus, this factor marks the starting point of the dark side of the SDT see [11].

There are different forms of motivation, the most autonomous forms include intrinsic motivation, integrated regulation and identified regulation. Intrinsic motivation refers to participating in the activity for the satisfaction and enjoyment inherent in it. Integrated regulation reflects that participation in activities is related to the “internal self”. Identified regulation implies that the behaviour is motivated by internal goals. Conversely, the least autonomous forms of motivation are introjected and external regulation, and somewhat apart, amotivation. These forms of motivation are caused by the frustration of basic psychological needs. Introjected regulation refers to participation in the activity to avoid feelings of guilt and shame. External regulation refers to participating due to social recognition and/or to obtain a prize. Amotivation refers to lacking initiative to participate in an activity due to the lack of apparent incentive, whether internal or external.

Different studies have analysed the consequences of the most autonomous forms of motivation regarding cognitive, affective, and behavioural well-being, and have found that there is a positive relationship [11,12]. Studies conducted by Standage, Gillison, Ntoumanis, and Treasure [13], and Trigueros, Aguilar-Parra, Cangas, López-Liria, and Álvarez [14] showed that high autonomous motivation favours adaptive behaviours at the behavioural level, which in turn favours the practice of PA outside of school hours, as well as healthy lifestyle habits. In contrast, low levels of autonomous motivation are related to maladaptation at the cognitive, behavioural, and affective levels.

Nonetheless, the models proposed by these and other studies did not measure the frustration of basic psychological needs in PE, so it is necessary to incorporate this variable in order to clarify the effects on the PE students’ motivation towards the subject. SDT can provide us with a motivational axis in which the social environment can have a series of consequences on the frustration and satisfaction of basic psychological needs and autonomous motivation, helping us to better understand how social influence may be related to motivational processes in the PE context, at the affective and cognitive levels.

### 1.2. Role of the PE Teacher

The role of the teacher during PE classes and his/her teaching style has been a very important element in various studies [15,16] as teachers play a relevant role that needs to be evaluated in order to measure the effectiveness and quality of the teaching and learning processes; this will result in student effectiveness and effort, which could lead to an improvement in the quality of the educational experience and in the students being motivated [17,18]. A considerable amount of research has been developed that has analysed the educational relationships between the teacher and his/her students based on a multitude of motivational theories.

In the context of the SDT, support for student autonomy by the teacher is a key teaching feature that encourages autonomous motivation in the educational context. Promoting personal initiative, self-regulation, self-concept, offering relevant objectives, and the use of minimum contingencies, will encourage support for student autonomy. On the other hand, a controlling style refers to the teacher’s use of external pressures, the use of coercive means, impositions, etc., which are perceived by students as the origin of their behaviour, undermining their own initiative, effort, and personal self-knowledge [19]. Most of the studies existing so far in the SDT field have analysed only how the teacher’s support for autonomy is related to student motivation by researching a series of adaptive behaviours. These studies have shown the positive relationship between support for autonomy and the satisfaction of basic psychological needs, autonomous motivation, and the performance of physical activity and sport outside school hours [20]. In addition, other studies have shown how support for autonomy has been negatively linked to the frustration of psychological needs and controlled motivation [14]. However, we have hardly found any studies analysing how the teacher’s use of a controlling interpersonal style, as perceived by PE students, affects the satisfaction and frustration of psychological needs and autonomous motivation, with the exception of the one conducted by Haerens et al. [11], the results of which were in line with the SDT postulates, given that the teacher’s controlling style was linked to controlled motivation and frustrated psychological needs whereas support for autonomy was linked to the satisfaction of psychological needs and autonomous motivation.

### 1.3. Mindfulness and Mind-Wandering in Education

Recently, a significant number of studies have incorporated mindfulness into education, with the aim of improving student well-being by acquiring greater self-awareness. The term mindfulness was popularized by Kabat-Zinn [21] who defined it as attention directed intentionally and without judgement, at the present moment taking place here and now. The first studies applying mindfulness programs were framed in terms of stress reduction and increased well-being in the work and life fields. A more current concept of this term was coined by Bishop et al. [22] defining it as the consciousness-centered awareness of the present, which does not elaborate and does not prosecute, in which the thoughts, emotions, or sensations that emerge in the attentional field are recognized and accepted as they are. In this way, contemporary psychology makes use of mindfulness in order to increase consciousness and respond skillfully to mental processes that generate emotional discomfort and behaviours that turn out to be maladaptive. Emotional discomfort and/or concerns can “enter” our minds while performing a certain activity, distracting us from it. As for mind-wandering, this involves loss of concentration or lack of awareness of the task being carried out, which prevents the assimilation of information [23]. Several studies claim that adolescents remain engrossed in their thoughts for around 50% of the time they spend in class, posing serious harm to their academic performance and personal development [24].

There are now numerous studies that have highlighted the effectiveness of techniques, therapies, and programs based on mindfulness [25,26]. This state can be developed using meditation, making us less reactive to what happens in the present moment [27]. A person who practices mindfulness acquires a greater knowledge of him/herself by accepting the thoughts, emotions, and sensations that are experienced in situ, becoming aware of their volatility and transient character, abandoning the pattern of judging them and considering them as elements with their own existence [28]. In addition to having a great influence on the emotions experienced (see [29]).

There are several studies in the educational field, both on teachers and on students, that have shown how mindfulness has been used successfully to reduce maladaptive conduct and behaviours (e.g., anxiety, depression), as well as improving their overall well-being [30]. Similarly, Franco, Soriano, and Justo [31] found significant improvements in student self-concept and academic performance. Gallego, Aguilar-Parra, Cangas, Rosado, and Langer [32] showed through a mindfulness intervention, increased academic performance, improved self-concept, and reduced anxiety in students.

However, there are hardly any studies in the academic field on mind-wandering. There is a study on reading comprehension where researchers provided a group of students with one easy and one difficult text to read [33]. During this exercise, the students were interrupted and asked whether the thoughts that invaded their minds at the time were related to reading or not. The conclusions of the study were that mind-wandering had a negative effect on understanding difficult texts. This is because tasks with a certain difficulty require great mental effort while simple tasks do not require as much, making the appearance of mental distractors possible.

In the PE context, we hardly have any record of studies on mindfulness and/or on mind-wandering, so we need to analyze the influence of both psychological elements on motivation and the adoption of active physical activity habits outside of school hours, given that the students’ full attention on themselves during PE classes is necessary for such adoption, as opposed to mind-wandering.

### 1.4. Emotions

In the educational field, emotions are a very important element in student academic performance since they are a highly significant motivational element, acting as triggers for the motivational processes inherent in the students [34]. An approximation of the term “emotions” is that proposed by Montañés [35] defining it as an evaluative assessment of an external situation that produces both psychological and physiological activation of the organism. Therefore, emotions are the result of a series of events and situations that are external, and that determine our actions.

In the educational field, it is believed that emotions can have a certain influence on students’ motivation to learn, study, and strive. This influence can affect the intrinsic motivation related to positive feelings, interest, and curiosity, as well as extrinsic motivation related to awards and external pressures. In addition, emotions can have an influence on the use of one or other learning strategies such as learning material design or memoristic learning. Various studies have found that positive emotions promote intrinsic motivation in a significant way, and to a lesser extent extrinsic motivation, which positively affects academic performance under most conditions. In contrast, negative emotions uniformly reduce motivation and information processing, which negatively affects performance [36]. As a result, emotions can have varying effects on students’ learning [37], although the negative effects on overall academic performance are likely to outweigh any beneficial consequences for most students [38].

However, the research that has taken place so far in the PE field is very fragmented, with theories and studies that address emotions individually (e.g., embarrassment [14]) or studies on the unique functions of emotions such as the Positive and Negative Affect Schedule (PANASN [39]) questionnaire, omitting the effect that different emotions can have on motivation, performance and, ultimately, on adopting healthy habits [14,40]. Despite this, there are several studies in the educational field of the primary stage where emotions have had a positive influence on motor competence, enthusiasm, and perception of Physical Education classes.

### 1.5. Objective and Hypothesis

For this study, the following hypotheses are posited: (1) Teacher support for student autonomy would positively predict the satisfaction of basic psychological needs (BPN) and, conversely, would negatively predict the frustration of BPN. For its part, the teacher’s controlling personal style would negatively predict the satisfaction of BPN yet positively predict the frustration of those needs; (2) the satisfaction of BPN would positively predict mindfulness and negatively predict mind-wandering, while frustration of BPN would negatively predict mindfulness and positively predict mind-wandering; (3) mindfulness would positively predict emotions while mind-wandering would predict them negatively; (4) positive emotions would positively predict autonomous motivation towards PE classes, PA, and the intention to be physically active while negative emotions would predict them negatively; (5) autonomous motivation towards PE classes would positively predict autonomous motivation towards PA classes; and finally (6) autonomous motivation towards PA classes would positively predict the intention to be physically active.

## 2. Method

### 2.1. Participants

This study involved 606 PE secondary school students, 323 boys and 283 girls (Table 1), aged between 13 and 19 (*M* = 15.7; *SD* = 1.43) from two secondary schools in Almeria (Spain). The inclusion criteria were voluntary participation in the study, the parents giving their informed consent (since the subjects were minors) and the students completing all of the questionnaires.

The completion of the questionnaires was carried out by those students who attended class that day, representing the 85.6% of the secondary school students.

### 2.2. Measures

Perceived support for autonomy. The Spanish version of the Perceived Autonomy Support Scale for Exercise Settings by Hagger and Chatzisarantis [41] was used, adapted by Moreno, González-Cutre, Chillón, and Parra [42] and validated to the Spanish PE context. This scale consists of 12 items that evaluate a single support for the autonomy factor. The instrument is scored on a Likert scale from 1 (totally disagree) to 7 (totally agree).

Psychological control. A version of the Psychologically Controlling Teaching Scale (PCTs [43]) was used, which was validated and adapted to the Spanish PE context by Trigueros, Aguilar-Parra, Cangas, and González-Santos [44]. The scale was preceded by the heading “My PE teacher...” and consisted of seven items (e.g., I feel guilty when I disappoint him/her) within a single factor. Students had to respond on a Likert scale ranging from 1 (totally disagree) to 5 (totally agree).

Satisfaction of basic psychological needs. A version of the Basic Psychological Needs in Physical Education Scale (BPN-PE; [45]) was used, validated, and adapted to the Spanish PE context by Menéndez and Fernández-Río [46]. To this scale we incorporated the items corresponding to novelty developed by González-Cutre et al. [9]. The scale is composed of a total of 18 items divided between four factors: Four items corresponding to autonomy, four items corresponding to competence, four items corresponding to each other, and six items corresponding to novelty. The responses were collected on a Likert-type scale ranging from 1 (totally disagree) to 7 (totally agree).

The frustration of basic psychological needs. The adapted version of the Frustration of Psychological Needs Scale was used with the incorporation of novelty (EFNPB; [47]), comprising the five items that make up the novelty factor. The scale was preceded by the heading “In my PE classes...” and consisted of 17 items, divided amongst each of the factors that make up the scale (e.g., autonomy, competence, relatedness, and novelty). Students had to respond on a Likert scale ranging from 1 (not true at all) to 7 (totally true).

Mind-wandering. A version of the Mind-w Questionnaire (MWQ; [48]) was used, which was validated and adapted to the Spanish PE context by Trigueros, Aguilar-Parra, Cangas, and Alvarez [49]. The scale was preceded by the heading “In my PE classes...” and consisted of five items (e.g., I do the exercises without paying attention) in a single factor. Students had to respond on a Likert scale ranging from 1 (almost never) to 6 (almost always).

Mindfulness during PE. A version of State Mindfulness Scale (SMS) questionnaire by Cox, Ullrich-French, Cole, and D’Hondt-Taylor [50] was used, validated, and adapted by Trigueros et al. [51]. This questionnaire was headed by the statement “During PE classes...”. It consisted of 12 items spread equally between two factors: Mental health (e.g., “I was aware that the thoughts come and go”) and physical or bodily experience (e.g., “I was aware of the different sensations in my body”). Subjects were required to indicate their response by means of a Likert scale from 1 (nothing at all) to 4 (a lot).

Emotions. The Scale of Emotional States in Physical Education validated by Trigueros, Aguilar-Parra, Cangas, and Alvarez [34] was used. The questionnaire is headed by the following statement “During PE classes...” comprising a total of 32 items divided among the eight factors that make up the scale (e.g., anxiety, shame, boredom, hopelessness, fun, pride, calmness, and trust). Students were required to respond using a Likert scale which ranged from 1 (totally disagree) to 7 (totally agree).

Motivation towards PE. In order to measure motivation towards PE classes, a version of the Perceived Locus of Causality-Revised (PLOC-R) by Vlachopoulos et al. [52] was used, validated, and adapted to the Spanish PE context by Trigueros et al. [53]. The scale was preceded by the heading “I participate in Physical Education class...” and consisted of 23 items grouped into six factors that measure intrinsic motivation, integrated regulation, identified regulation, introjected regulation, external regulation, and amotivation. The students responded using a Likert scale ranging from 1 (not true at all) to 7 (totally true).

To evaluate autonomous motivation, the Self-determination Index (SDI-PE; [54]) was used, calculated from the following formula: 3 × intrinsic motivation, 2 × integrated regulation, 1 × identified regulation, −1 × introjected regulation, −2 × external regulation, and −3 × amotivation. This index has proven valid and reliable in several works. It is used to obtain a value for quantifying the level of self-determination.

Motivation towards PA. The Behavioural Regulation in Exercise Questionnaire (BREQ-3) by Wilson, Rodgers, Loitz, and Scime [55], validated and adapted to the Spanish context by González-Cutre, Sicilia, and Fernández [56], was used to measure the motivation that practitioners of physical exercise possessed. This questionnaire consists of 23 items related to intrinsic regulation spread across six factors: Intrinsic motivation, integrated regulation, identified regulation, introjected regulation, external regulation, and amotivation. Students responded using a Likert scale ranging from 0 (Not true at all) to 4 (Totally true), which was headed by the statement “I practice physical exercise...”.

To evaluate autonomous motivation, the Self-determination Index (SDI-PA; [54]) was employed, calculated from the following formula: 3 × intrinsic motivation, 2 × integrated regulation, 1 × identified regulation, −1 × introjected regulation, −2 × external regulation, and −3 × amotivation. This index has proven valid and reliable in several works. It is used to obtain a value for quantifying the level of self-determination.

Intentionality of being physically active. A version of the Intention to be physically active scale by Hein, Müür, and Koka [57] was used, adapted, and validated to the Spanish PE context by Moreno, Moreno, and Cervelló [58]. This scale consists of five items to measure a single factor. The items are preceded by the phrase “Regarding your intention to practice some physical/sports activity...”. The responses correspond to a Likert scale that ranges from 1 (totally disagree) to 5 (totally agree).

### 2.3. Procedure

To carry out the study, the various schools in the province of Almería were contacted previously to request the necessary permission to give the questionnaires to their students. At the same time, they were informed of the study objectives. Before administering the scales to all participants, the parents or tutor were asked to fill in and sign informed consents, as the subjects were minors. The questionnaire was administered under the supervision of an expert survey taker, a member of the research group, who explained the procedure and resolved any doubts that arose while the students completed it. The time estimated to complete the questionnaires was around 25 min.

This study was carried out in accordance with the recommendations of the American Psychology Association. The entire experiment was conducted in accordance with the Helsinki Declaration. Ethics approval was obtained from the Research Ethics Committee of the University of Almeria, Spain (Ref. UALBIO 2019/014).

### 2.4. Data Analysis

First, the descriptive statistics were calculated and, using the Pearson correlation, a correlation analysis was performed between the study variables. Subsequently, the hypothesized predictive model was tested using a structural equation model (SEM). To check the mediation effects between the model variables, the premises established by Baron and Kenny [59] were taken into account: (a) Significant correlations between the independent and dependent variables; (b) significant correlations between the independent variable and mediators; (c) significant correlations between the mediators and the dependent variable; and (d) the previous significant relationship between the independent variable and the dependent variable is no longer significant when the relationships between the independent variable and the mediators, and between them and the dependent variable, are controlled.

For the SEM, the maximum likelihood estimation method was used with the bootstrapping procedure in the AMOS 21 statistical package. This procedure revealed the robustness of the estimations [60]. The following indices were used to analyze the model’s goodness of fit: The χ^2^ coefficient, the chi-square divided degrees of freedom (χ^2^/df), the comparative fit index (CFI), the incremental fit index (IFI), the root mean square error of approximation (RMSEA) plus its 90% confidence interval, and the standardized root mean square residual (SRMR). Typically, χ^2^/df values of less than 5 [61], CFI and IFI values equal to or greater than 0.90, values of 0.08 or lower for RMSEA, and 0.06 or lower for SRMR [62] were considered acceptable. However, Marsh, Hau, and Wen [63] stated that these cut-off values should be interpreted with caution, as they can be too restrictive and difficult to achieve when testing complex models.

## 3. Results

### 3.1. Preliminary Analysis

As can be seen in Table 2, the descriptive statistics and correlations between the study variables appear. Likewise, the reliability analysis using Cronbach’s alpha yielded the following values: 0.81 for Psychological Control, 0.84 for Autonomy for Support, 0.87 for Frustration of Psychological Needs, 0.78 for Satisfaction of Psychological Needs, 0.82 for Mind-wandering, 0.79 for Mindfulness, 0.75 for Positive Emotions, 0.80 for Negative Emotions, and 0.89 for PA Practice Intention.

### 3.2. Structural Equations Model

**Figure 1:**
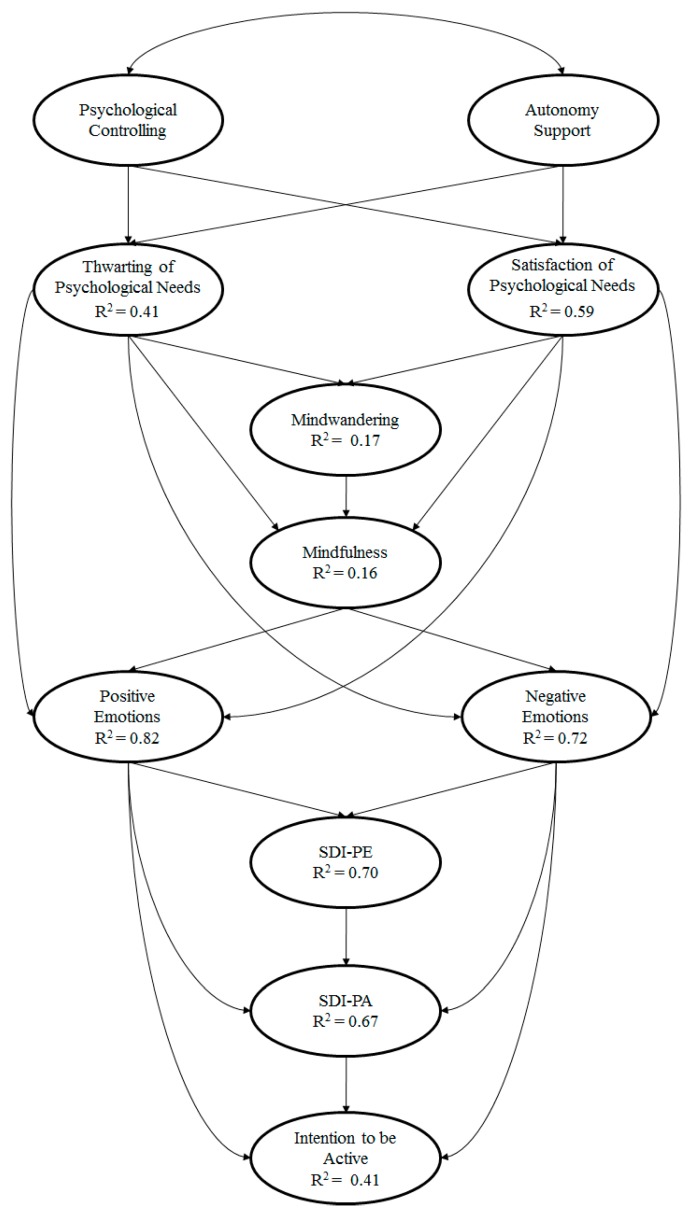
Hypothesized model of the educational context.

Before testing the hypothesized model using an SEM and analysing the relationships between the study variables, the number of latent variables was reduced as each had at least two indicators, given the model’s complexity [64]. The hypothesized predictive relationship model (Figure 1) showed the following fit indices: χ^2^ (381. N = 606) = 1634.89, p < 0.001; χ^2^/df = 4.29; CFI = 0.92; IFI = 0.92; RMSEA = 0.075 (IC 90% = 0.073–0.080); SRMR = 0.047. The following describes the relationships obtained between the different factors that make up the model:

(a) The correlation between psychological control and support for autonomy was negative (β = −0.59, *p* < 0.001).

(b) Psychological control positively predicted the frustration of psychological needs (β = 0.53, *p* < 0.001) and, in turn, negatively predicted the satisfaction of psychological needs (β = −0.17, *p* < 0.01).

(c) Support for autonomy positively predicted the satisfaction of psychological needs (β = 0.66, *p* < 0.001) and, in turn, negatively predicted the frustration of psychological needs (β = −0.16, *p* < 0.001).

(d) The satisfaction of psychological needs negatively predicted mind-wandering β = −0.20, *p* < 0.01) and negative emotions β = −0.54, *p* < 0.001). However, it positively predicted mindfulness (β = 0.34, *p* < 0.001) and positive emotions (β = 0.82, *p* < 0.001).

(e) The frustration of psychological needs positively predicted mind-wandering (β = 0.30, *p* < 0.001) and negative emotions (β = 0.47, *p* < 0.001). However, it negatively predicted mindfulness (β = −0.12, *p* < 0.05) and positive emotions (β = −0.12, *p* < 0.001).

(f) Mind-wandering negatively predicted mindfulness (β = −0.14, *p* < 0.01).

(g) Mindfulness positively predicted positive emotions (β = 0.22, *p* < 0.05) and, in turn, negatively predicted negative emotions (β = −0.12, *p* < 0.05).

(h) Positive emotions positively predicted SDI-PE (β = 0.81, *p* < 0.001), the SDI-PA (β = 0.20, *p* < 0.001), and the intention to be physically active (β = 0.41, *p* < 0.001), while negative emotions negatively predicted the SDI-PE (β = 0.15, *p* < 0.01) SDI-PA (β = −0.11, *p* < 0.001) and the intention to be physically active (β = −0.21, *p* < 0.01).

(i) The SDI-PE positively predicted SDI-PA (β = 0.77, *p* < 0.001) and finally, the SDI-PA positively predicted the intention to be physically active β = 0.27, *p* < 0.001).

## 4. Discussion

Through this study we have tried to analyze the relationships between the teacher’s interpersonal style regarding psychological needs (satisfaction and frustration), mind-wandering, mindfulness, positive and negative emotions, autonomous motivation for PE classes and PA, and the intention to be physically active, in secondary school students.

For the first time in Spain, this study looks at the role of the teacher from the duality of support for autonomy compared to a controlling style, and relating both these styles to the frustration and satisfaction of psychological needs, again seen for the first time in a study concerning the field of PE classes. However, there are similar studies from other countries that have linked the interpersonal style of the teacher from the control compared to support for autonomy duality as opposed to the psychological needs of frustration and satisfaction, showing the relevance of the teacher in the social and psychological development of the students [11,65]. We also looked at the role of the students’ psychological needs, since these are considered as basic and universal supports present in all human beings, which can have a series of positive or negative consequences at the psychological and behavioural levels, depending on whether they are satisfied or not [66].

Various studies in the PE field have noted the positive effect of support for autonomy in relation to the satisfaction of psychological needs in PE students, and this, in turn, on motivation towards such classes [67,68]. Nevertheless, despite this evidence, the negative aspects present during PE classes are barely understood, such as the teacher’s controlling style and the frustration of psychological needs—depending on the teacher’s role and the students’ perception of the PE classes, they can have a negative influence on adopting future behaviours towards PA practice or adopting healthy lifestyle habits.

A study carried out by Haerens et al. [11] showed how the perception of support for autonomy and the control of teaching, as well as the satisfaction of psychological needs and the frustration of psychological needs, constitute different constructs that are related specifically to motivational results. The perceived support for autonomy was mainly related to autonomous motivation, with the satisfaction of psychological needs being a mediator to this association, while the perception of controlling teaching style was mainly related to controlled motivation and amotivation, acting as a mediator of frustration. The above results are in accordance with the results from our own study, where support for autonomy positively predicted the satisfaction of psychological needs and negatively predicted the frustration of psychological needs; conversely, psychological control positively predicted the frustration of psychological needs and negatively predicted the satisfaction of psychological needs.

Accordingly, this study seeks to resolve certain limitations that are generally found in the studies so far carried out internationally, and in particular in Spain, which we have tried to take into account, showing a model where both interpersonal relationships of the teacher are related to the satisfaction and frustration of psychological needs. Thus, the results obtained are in line with those shown in previous studies and with the SDT postulates. The results between the dual role of the teacher and the psychological needs established in this study can be explained in such a way that, if students perceive a certain freedom of execution and decision-making ability, this will benefit their perceived competence, their psychological well-being, and the satisfaction of their psychological needs. However, if the teacher behaves in an autocratic, restrictive, or forceful way towards the students, they will feel oppressed, incapable, and rejected, tending to feel that their psychological needs are thwarted.

The results also showed that the satisfaction of psychological needs positively predicted mindfulness and positive emotions while negatively predicting mind-wandering and negative emotions. In contrast, the frustration of psychological needs negatively predicted mindfulness and positive emotions while positively predicting mind-wandering and negative emotions. These results cannot be compared to previous studies, as this is the first time that the relationship between psychological needs and previous study variables have been contemplated in the PE context. Nonetheless, various authors [69,70] highlight the importance of psychological needs in generating adaptive and maladaptive behaviors in humans, depending on whether these needs are satisfied or frustrated. The possible reason for this is that, if the person feels a certain satisfaction of his/her psychological needs, this would entail the integral development of his/her own physical and cognitive abilities, also adopting an active role in society. A study on HIV patients demonstrated that those who had high satisfaction of psychological needs presented high levels of mindfulness, sleep quality, and quality of life [28]. Similarly, a study by Wei, Shaffer, Young, and Zakalik [71] showed how the satisfaction of psychological needs led to a negative relationship with negative emotions, such as depression, loneliness, shame, and attachment. On the other hand, a study carried out by Alexander [72], in which the study population was made up of adolescents who engaged in playing video games, demonstrated amongst other things that the satisfaction of psychological needs showed a positive relationship regarding emotions such as fun, pleasure, and pride with respect to electronic games. Thus, it has been observed that psychological needs play a fundamental role, not only in people’s emotional, social, and cognitive development, but also in adopting certain behaviours that, as stated previously, can be adaptive or maladaptive. In addition, the results have shown how mindfulness has positively predicted negative emotions and negatively predicted negative emotions. These results cannot be compared with similar studies in the field of Physical Education classes, however from the field of primary education a study conducted by Meiklejohn et al. [73] showed how those students who developed a high level of mindfulness showed a greater emotional regulation and therefore a greater predisposition towards positive emotions during classes. Therefore, the results obtained in the present study can be explained by the fact that those students who have developed a greater mindfulness generate strategies of acceptance of the emotions experienced with joy of positive emotions, however the negative emotions are seen as undesirable, one tries to get rid of them in one way or another, which is often unsuccessful, generating an increase in negative emotions, thus having a high level of mindfulness is being aware of these negative emotions, accepting them as they are, and being simpler to get rid of them.

On the other hand, the results showed that positive emotions positively predicted autonomous motivation towards physical education classes, towards physical activity, and the intention to be physically active; whereas negative emotions negatively predicted autonomous motivation towards physical education classes, physical activity, and the intention to be physically active. Nevertheless, these results are difficult to compare with other studies because research on emotions in education is relatively fragmented [74]. Accordingly, a study conducted by Mega, Ronconi, and De Beni [75] showed the influence of emotions on various facets of motivation towards learning. In particular, students’ positive emotions improve their beliefs in their own abilities and the intention to perform certain actions. In addition, positive emotions exerted a greater weight on motivation than did negative emotions. These results highlighted and demonstrated the relevance of positive emotions on motivation towards learning, distinguishing the premise already established by Pekrun et al. [74], that positive emotions should be worked on and enhanced in academic environments to promote student motivation and learning.

In the same vein, the results of this study have shown the predictor role that emotions towards PE classes have regarding autonomous motivation towards PE classes and towards physical activity and the intention to be physically active, showing that positive emotions exert a greater weight than negative emotions. Emotional stimuli interact to some extent with cognitive processes affecting reasoning ability, memory, attitude, disposition and interest, aspects that are all linked to motivation and intention.

Finally, the results have shown the relationship between autonomous motivation towards PE classes and towards physical activity and the intention to be physically active. They are in accordance with the postulates of self-determination theory and with numerous studies [76,77]. They support the idea of a synergy between PE classes and physical activity—if the student experiences an increase in motivation towards PE classes, this could generate the necessary motivation to encourage their commitment to stay physically active in and out of school, resulting in a life-long commitment [78].

In short, the SDT postulates have been supported by the introduction of new variables, showing their applicability to Spanish culture. However, in regard to the model’s findings, one should emphasize that this is a correlational study and so it does not allow us to extrapolate cause–effect relationships; also, the results could be interpreted in multiple ways depending on how each person understands them. The study has sought to expose possibilities rather than causality so we can explain the relationships between the variables in both studies. The model seems to demonstrate good robustness and a capacity to be generalised towards different cultures or age groups, helping us to understand a little better the role that the teacher plays in consolidating healthy lifestyle habits. In addition, future studies should analyze the influence of the social context (i.e., parents, friends) on the involvement of students in EF classes.

## Figures and Tables

**Table 1:** Sociodemographic characteristics.

	*N*
Male	323
13–15y	148
16–17y	94
18–19y	81
Female	283
13–15y	126
16–17y	83
18–19y	74

**Table 2:** Descriptive statistics and correlation between variables.

Variables	*M*	*SD*	1	2	3	4	5	6	7	8	9	10	11
1. Psychological Control	1.84	1.09		−0.57 **	−0.39 **	0.47 **	−0.41 **	−0.29 **	−0.53 **	0.50 **	−0.42 **	−0.79 **	−0.54 **
2. Support for autonomy	4.47	1.34			0.52 **	−0.38 **	−0.33 **	0.28 **	0.60 **	−0.47 **	0.48 **	0.64 **	0.59 **
3. SPN	4.44	1.64				−0.35 **	−0.30 **	0.29 **	0.68 **	−0.54 **	0.58 **	0.69 **	0.66 **
4. TPN	2.72	1.47					0.32 **	−0.15 **	−0.42 **	0.47 **	−0.42 **	−0.63 **	−0.49 **
5. Mind-wandering	2.35	1.29						−0.26 **	−0.35 **	0.37 **	−0.30 **	−0.41 **	−0.42 **
6. Mindfulness	3.26	0.72							0.36 **	−0.28 **	0.32 **	0.36 **	0.36 **
7. Positive Emotions	4.87	1.47								0.58 **	0.65 **	0.77 **	0.76 **
8. Negative Emotions	2.56	1.53									−0.65 **	−0.72 **	−0.85 **
9. SDI-PE	11.40	16.09										0.85 **	0.76 **
10. SDI-PA	7.60	11.03											0.70 **
11. Intention to be Active	3.79	1.28											

** *p* < 0.01. Note: FPN: Thwarting of psychological needs; SPN: Satisfaction of psychological needs; SDI-PE: Self-Determination Index towards Physical Education; SDI-PA: Self-determination Index towards Physical Activity.
